# Supplementary material for: H3K27me3 Depletion during Differentiation Promotes Myogenic Transcription in Porcine Satellite Cells
Source: Genes (Basel). 2019 Mar 19;10(3):231. doi: 10.3390/genes10030231 (PMC6471710; doi:10.3390/genes10030231)
Supplement: Supplementary file 1 [file genes-10-00231-s001.pdf]

Figure S1.

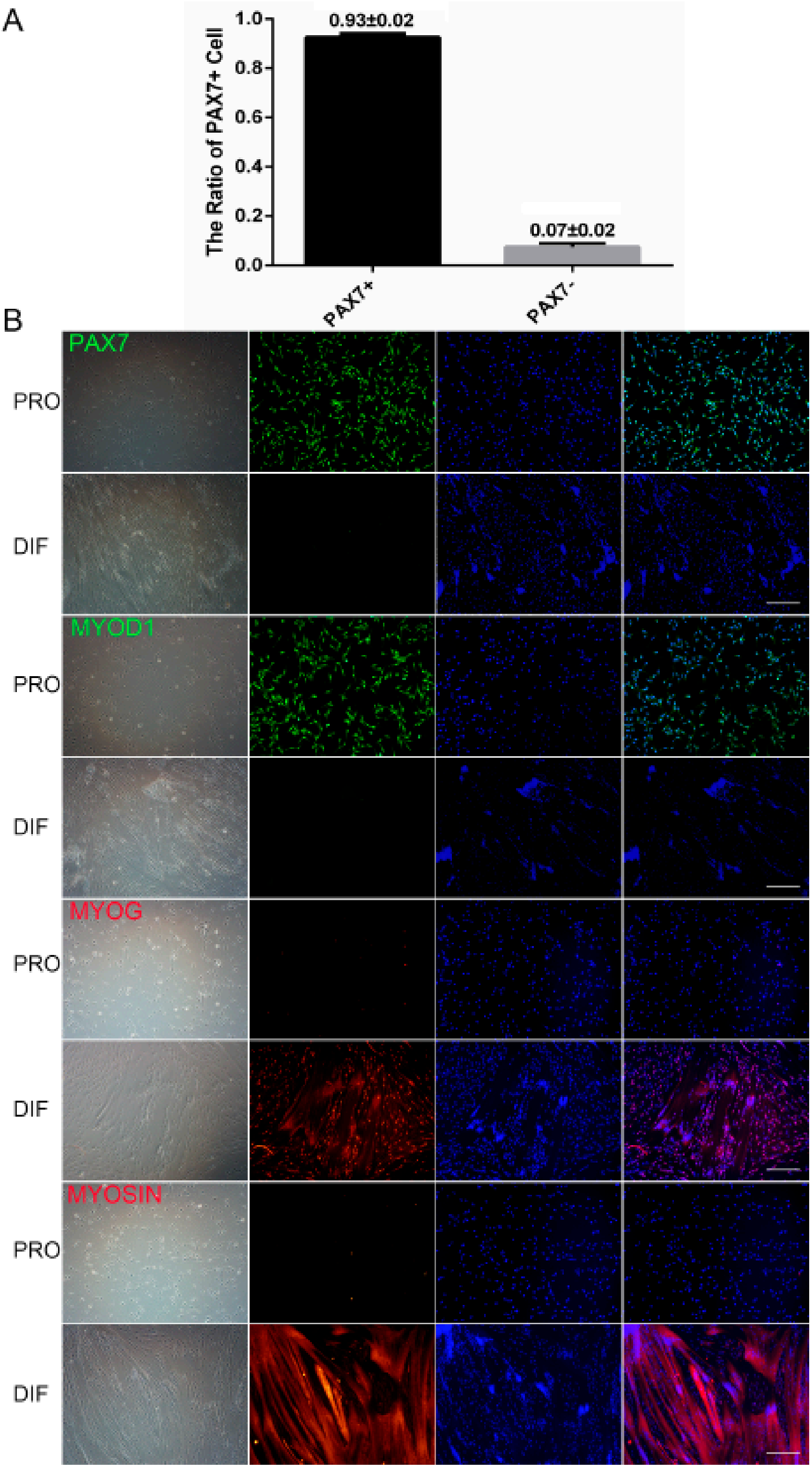

Figure S1. Porcine satellite cells (PSCs) culture and immunofluorescence detection. (**A**) Statistical analysis on the purity ratio of PAX7+ PSCs. The purity ratio of PAX7+ cells was calculated using immunofluorescent staining, which was presented as mean ± SEM (five random fields are captured for each group). (**B**) Immunofluorescence detection of proliferation cells (PRO, upper panel) and 2-day differentiated cells (DIF, bottom panel). Antibodies (top to bottom: PAX7, MYOD1, MYOG, MYOSIN) were indicated as green and red, Hoechst33342 dye was blue. Scale bars: 200 μm. Magnification: 100×.

Figure S2.

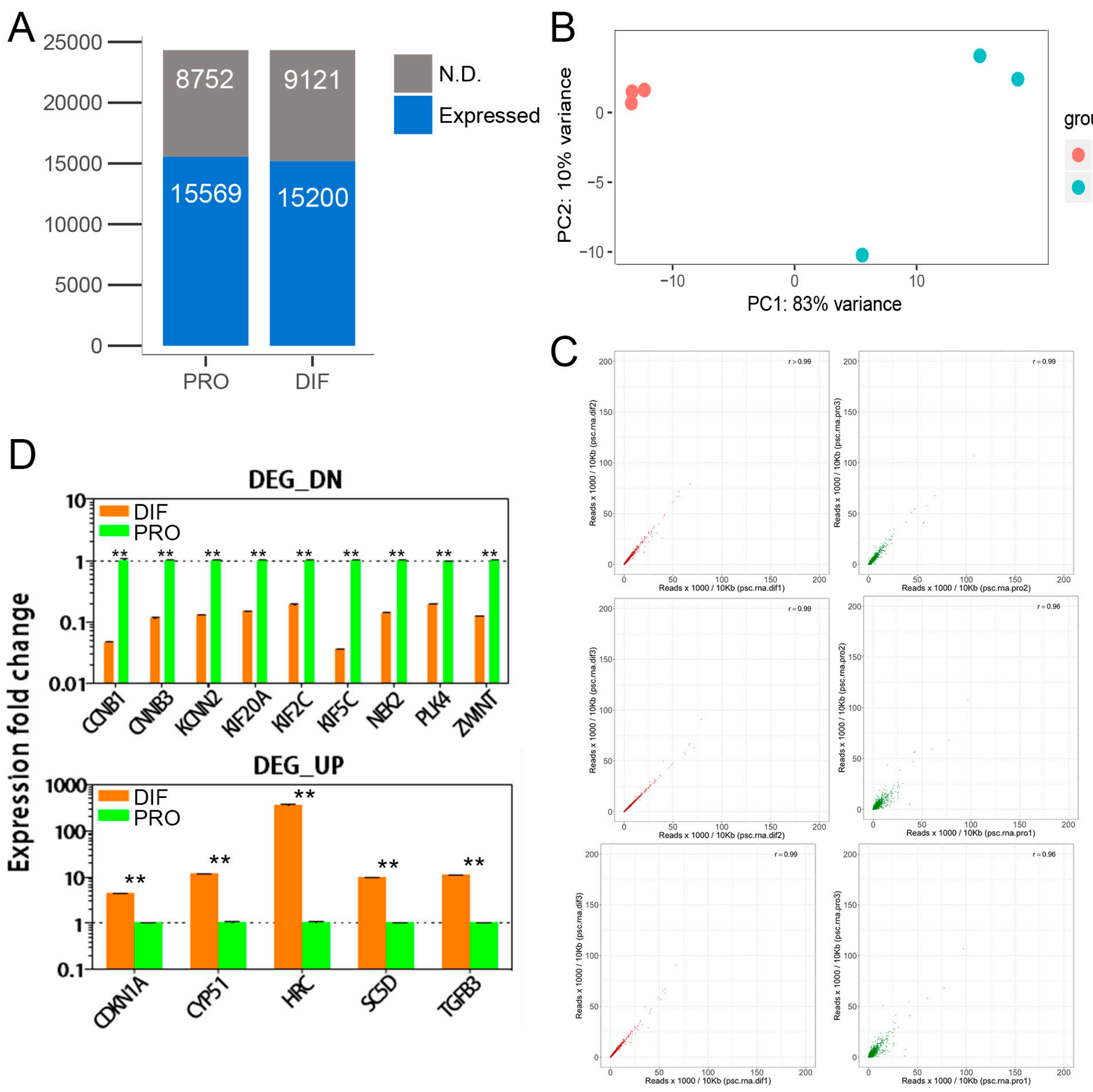

Figure S2. Transcriptome profiles exhibited using RNA-seq. (**A**) The number of genes detected in PRO and DIF cells. Genes were split into expressed (Expressed) and not detected (N.D.) parts. (**B**) Principal component analysis (PCA) of transcriptomes of PRO and DIF cells (three biological replicates for each). The first and second principal components were depicted as x-axis and y-axis. (**C**) The correlationships between three replicates of transcriptome profiles of PRO and DIF cells were illustrated. (**D**) Quantitative PCR (qPCR) was performed to validate the differentially expressed genes (DEGs) changed during cell differentiation. Nine down regulated and five upper regulated genes were selected. Three replicates were performed, and the results were presented as mean ± SEM; ***p* <0.01.

Figure S3.

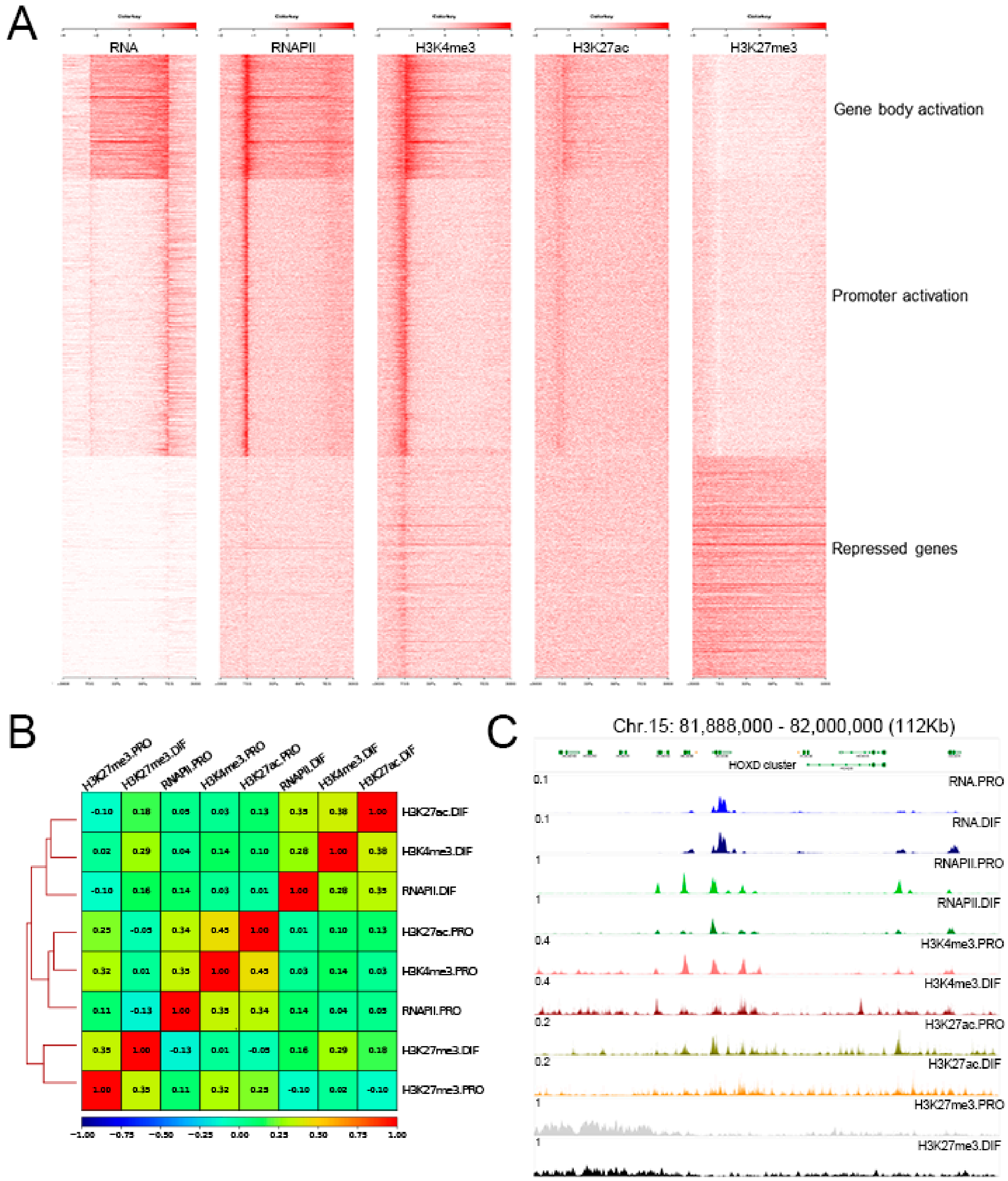

Figure S3. Epigenetic profiles of PRO and DIF cells. (**A**) Heatmap was used to plot Chromatin Immunoprecipitation Sequencing (ChIP-Seq) enrichment, which illustrated the modification of three histone modifications and RNA polymerase Ⅱ(RNAPII) and the correlation associated with gene expression profiles in PRO cells. Maps were generated from 3 kb upstream of the transcription start site (TSS) to 3 kb downstream of the transcription end site (TES). (**B**) Heatmap was used to show the correlation between three epigenetic marks and RNAPII during cell differentiation using spearman model. (**C**) The integrative genomics viewer (IGV) browser views showing the ChIP-Seq and RNA-Seq enrichment between PRO and DIF cells, the *HOXD* cluster was captured to show the fractions of peaks of three epigenetic marks and RNAPII.

Figure S4.

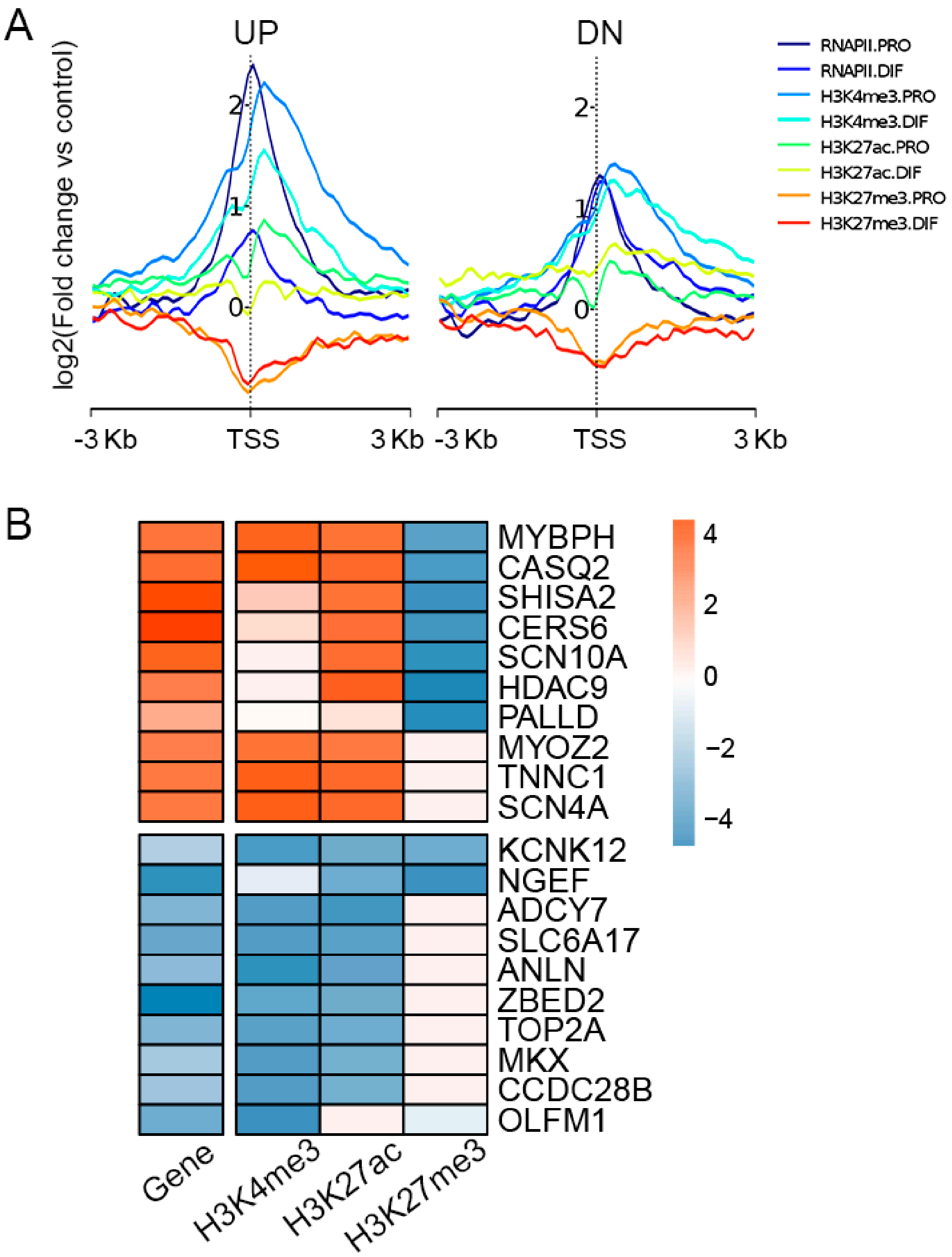

Figure S4. The relationships between epigenetic modifications and gene expression during cell differentiation. (**A**) Expression profiles of 917 DEGs of three histone modifications and RNAPII were plotted between ± 3 kb of TSS region during differentiation. Up and down regulated DEGs were described separately. The y-axis was the average log2 fold change compared with control. (**B**) Heatmap illustrating the relationships of the changes of histone modifications and gene expression of 20 DEGs of PRO and DIF cells. Gene expression and histone modifications were clustered using log2 fold change of RPKM.

Figure S5.

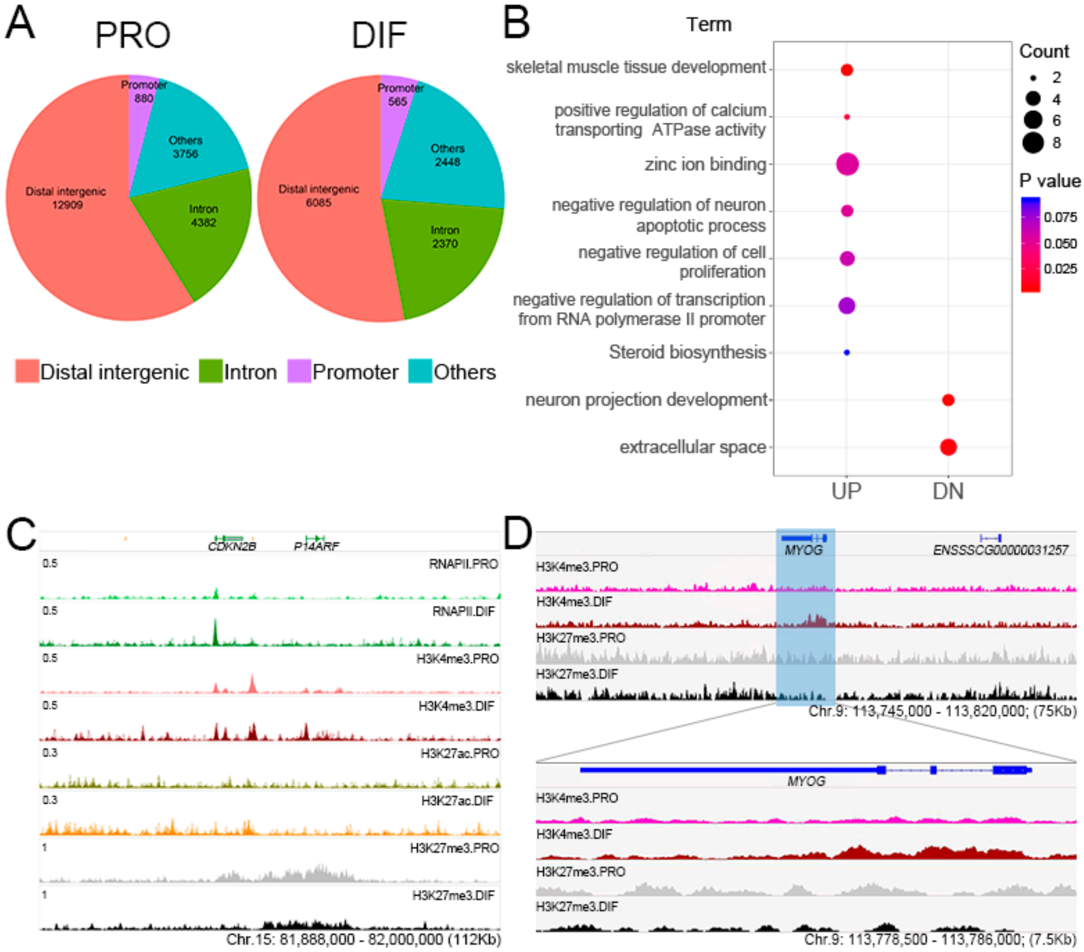

Figure S5. Gene Ontology (GO) analysis of H3K27me3 modified DEGs. (**A**) The intersection sizes were illustrated using pie charts to show the distinction of H3K27me3 modification during cell differentiation. (**B**) The functions of the upper (UP) and down (DN) regulated DEGs were illustrated, where nine pathways and biological processes were demonstrated. (**C**) The IGV browser was used to show the ChIP-Seq enrichment for *CDKN2B*, which was released from H3K27me3 histone modification during cell differentiation. (**D**) The TSS region of MYOG was illustrated using IGV browser to show the depletion of H3K27me3 during cell differentiation.

Figure S6.

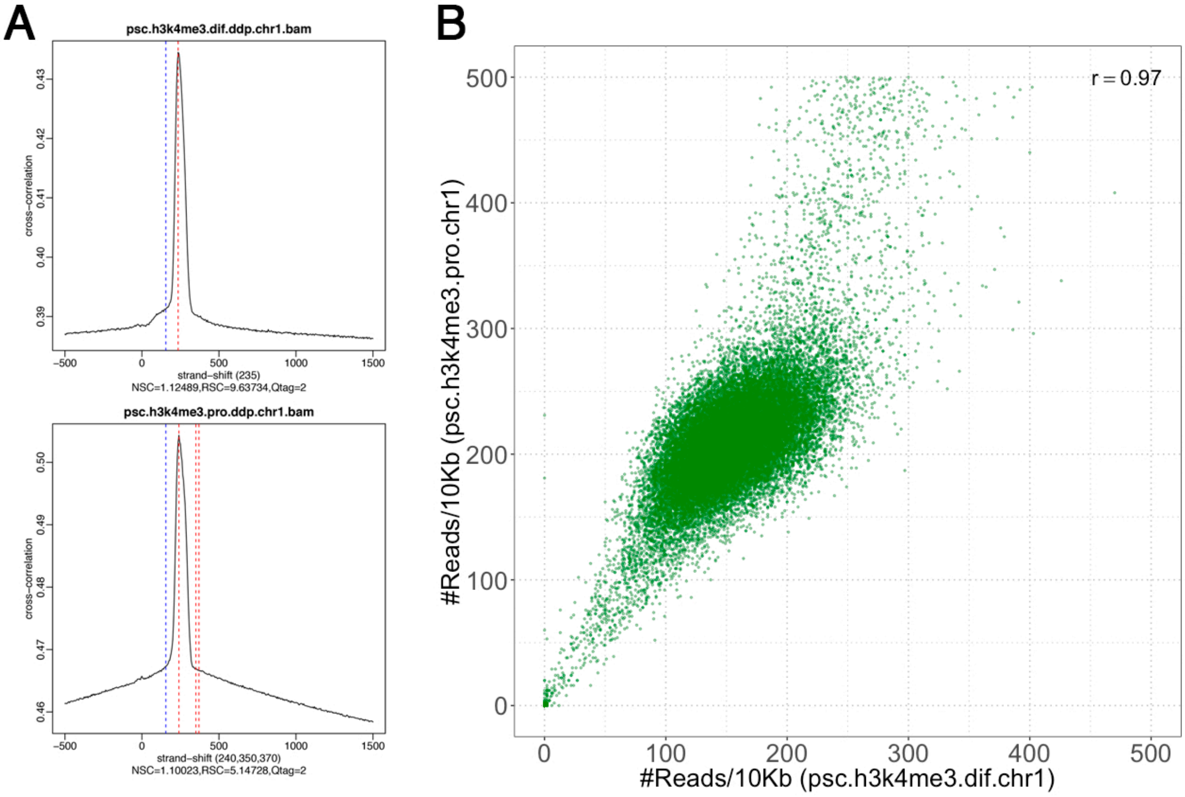

Figure S6. Data reproducibility of H3K4me3 on Chr 1. (**A**)The cross-correlation of DIF (top) and PRO (bottom) indicates they have a highly consistent position and length (~240 bp) of phantompeak. (**B**) The peak reproducibility also showed that DIF and PRO are highly correlated (r = 0.97) based on the peak reads per 10 Kb length.
